# Optimal MHC-II-restricted tumor antigen presentation to CD4+ T helper cells: the key issue for development of anti-tumor vaccines

**DOI:** 10.1186/1479-5876-10-154

**Published:** 2012-07-31

**Authors:** Roberto S Accolla, Giovanna Tosi

**Affiliations:** 1 Department of Surgical and Morphological Sciences, University of Insubria, Via Ottorino Rossi, n.9, 21100 Varese, Italy

**Keywords:** Tumor immunity, Vaccine, T helper cells, CIITA, MHC class II

## Abstract

Present immunoprevention and immunotherapeutic approaches against cancer suffer from the limitation of being not “sterilizing” procedures, as very poor protection against the tumor is obtained. Thus newly conceived anti-tumor vaccination strategies are urgently needed. In this review we will focus on ways to provide optimal MHC class II-restricted tumor antigen presentation to CD4+ T helper cells as a crucial parameter to get optimal and protective adaptive immune response against tumor. Through the description of successful preventive or therapeutic experimental approaches to vaccinate the host against the tumor we will show that optimal activation of MHC class II-restricted tumor specific CD4+ T helper cells can be achieved in various ways. Interestingly, the success in tumor eradication and/or growth arrest generated by classical therapies such as radiotherapy and chemotherapy in some instances can be re-interpreted on the basis of an adaptive immune response induced by providing suitable access of tumor-associated antigens to MHC class II molecules. Therefore, focussing on strategies to generate better and suitable MHC class II–restricted activation of tumor specific CD4+ T helper cells may have an important impact on fighting and defeating cancer.

## 

I asked a bird if he were happy

Give me more sky, was the answer

I asked a fish if he were happy

Give me more ocean, was the answer

I asked myself the same question

Give me more birds and more fishes, was the answer

(***anonymous african poet)***

## Background

Although the host can mount an immune response against cancer cells [1] the fact that the tumor takes off in cancer patients demonstrates that tumor may elude immune defences [1,2]. Tumors are not simple entities. They are composed of tumor cells, tumor stroma and often of a series of blood-derived infiltrating leukocytes including cells of innate and adaptive immunity [3]. It has become apparent that tumor-infiltrating leukocytes, including neutrophils, macrophages, mast cells, eosinophils [4,5] as well as T cells with CD4+/CD25+ phenotype and suppressive function on helper and effector T cells, designated regulatory T cells (Tregs) [6,7], can cooperate in favouring, instead of antagonizing, tumor growth. These findings have created a diffuse sentiment that a sort of pro-tumor polarization of the innate and adaptive immunity is the cause for tumor cells to survive, replicate and spread [8,9].

As seen from the side of the protective adaptive immune response, however, the above events can be interpreted not as the cause but simply as the consequence of the tumor strategy to primarily counteract components of the acquired immunity (see Table 1). Thus, the intention here is to re-establish the role of the acquired immune response (that is: specific antigen presentation, stimulation of antigen-specific CD4+ T helper (TH) cells and generation of antigen-specific effector cells) as the major mechanism of defence against cancer. Instrumental for this role is to reach optimal MHC class II (MHC-II)-dependent tumor antigen presentation for triggering tumor-specific TH cells and, by this, the downstream cascade of adaptive anti-tumor immunity to counteract not only tumor onset but also established tumors. These considerations have important consequences also for the comprehension of how chemotherapy and/or radiation therapies may help to block and/or to eradicate the tumor and for the construction of suitable anti-tumor vaccine strategies.

**Table 1:** Major elements influencing tumor escape from adaptive immune recognition and destruction

**1. Tumor-related**
- Insufficient tumor antigen expression
- Loss of MHC class I expression by tumor
- Lack of MHC class II expression by tumor
- Production of immunosuppressive factors for T and B cells
**2. Immune cells-related**
- Insufficient lymphocyte penetration into the tumor tissue
- Lack of T cell help
Insufficient MHC-II-restricted antigen presentation
Insufficient TH triggering
- Lack or insufficient CTL activity
Insufficient MHC-I-restricted antigen presentation
Insufficient support by TH cells
Scarce lytic activity
- Extrinsic functional blocking of T cells
Regulatory and suppressor cells
Inhibitory cytokines

## Inefficiency of immune effectors in tumor-bearing hosts

Often immune cells with specificity for tumor-associated antigens can be found in cancer patients. Due to the justified belief, based on the groundbreaking studies of Festeinstein [10], that the major mechanism of elimination of tumor cells should rely on MHC class I (MHC-I)-restricted CD8+ cytolytic T lymphocytes (CTL), most tumor immunologists focussed initially their attention on the presence of CTL in tumor-bearing hosts. Indeed, CTL can be isolated from tumor tissues, their fine tumor antigen specificity can be assessed, they can be amplified in vitro and re-injected into hosts in which, at least in some experimental animal models, they can inhibit tumor take or even cure established tumors [11]. On this basis, clinical trials using CTL-defined antigens as vaccines have been performed. However, in most studies the CTL responses were weak and unable to control tumor growth and metastasis [12]. This event was due not only to the frequent loss or reduced expression of MHC-I molecules in tumor cells [13-15] but also to the poor tumor specific, MHC-II-restricted T cell help generated in tumor-bearing patients [16], as TH cells are required for optimal induction of both humoral and cellular effector mechanisms [17] and particularly for CTL maturation, clonal expansions and acquisition of cytolytic function [18].

TH cell triggering requires recognition of antigenic peptides presented by MHC-II molecule expressed on professional antigen presenting cells (APC) including dendritic cells (DC), macrophages and B cells [19]. Inhibiting and/or avoiding the crucial phase of MHC-II-dependent tumor antigen presentation to and/or activation of TH cells would thus be an effective strategy to block *ab origine* the adaptive anti-tumor immune response.

Among the possibilities to explain the insufficient/absent MHC class II-dependent stimulation of tumor associated antigen-specific TH cells the following events have been hypothesized:

1. *Tumors elaborate insufficient amounts of tumor-associated antigens:* scarcity of necrotic and/or apoptotic tumor cells to serve as potential source of exogenous antigens in fuelling antigen presentation would hamper the power of presenting immunologically relevant amounts of TAA to TH cells, even if APC are functionally available.

2. *Tumor cells elaborate products that actively inhibit the function of APCs:* tumor cell may secrete cytokines and chemokines that are inhibitory for either antigen uptake, processing or MHC-II-dependent presentation by APC.

3. *Tumor cells may not be suited for acting as surrogate APC:* tumor cells are not classical APCs and they usually do not express MHC-II molecules. This prevents their possibility of functioning as surrogate APC for their putative TAA.

4. *Low frequency of TH cells against tumor-associated antigens:* because tumor antigens are mostly self constituents and very often non mutated products, the frequency of TH cell precursors specific for MHC-II-restricted TAA peptides may be very low and unable to guarantee an efficient effector function upon antigen stimulation.

## Providing optimal MHC-II-restricted tumor antigen presentation to TH cells: a key parameter for an efficient anti-tumor immunity

A crucial parameter for TH activation is the optimal MHC-II-restricted antigen presentation and related antigen processing capable of efficiently triggering tumor-specific TH cells. Intentionally, we do not specify the nature of the antigen presenting cells because, as it will appear later, this role could be played, at least in part, by the tumor cells themselves. Based on recent results of our group and of other investigators, along with reappraisal of historical seminal experiments in immunology, we will underline the notion that providing “more antigen availability” under the form of optimal MHC-II-restricted antigen presentation to TH cells is a key element for generating the cascade of immune effector functions and polarizing signals that result in tumor rejection and acquisition of anti-tumor immune memory.

Fifty years ago Mitchison demonstrated the importance of antigen dose in triggering an adaptive immune response [20,21] by showing that low amounts of protein antigen would never trigger an immune response and might even be tolerogenic. In a recent adaptation of original Mitchison’s paradigm of “more antigen-more response” Zinkernagel and coworkers elaborated the theory that for tumor antigens not only the quantity, but also the “geographic availability” is a crucial parameter to instruct the adaptive immune system [22]. If cells of adaptive immunity do not have the chance to encounter tumor cells because the tumor remains localized at sites out of contact with immune cells, than the tumor will be neglected, “ignored” by the immune system.

## Optimal MHC-II-restricted antigen presentation to TH cells in preventive anti-tumor vaccination: the example of CIITA-induced MHC class II expression in tumor cells

In the recent past, many attempts have been made to construct preventive anti-tumor vaccines by using as a primary source of antigen whole tumor cells treated by different procedures to make them more immunogenic. Irradiated or genetically modified tumor cells have been used even in clinical trials [23].

As a different approach to obtain optimal triggering of the adaptive anti-tumor immune response, a vaccination strategy with tumor cells transduced with the *AIR-1*-encoded MHC-II transactivator CIITA [24,25] has been explored in our laboratory. The rationale underlying this approach was that CIITA-transfected tumor cells may act as “surrogate APC” *via* MHC-II-restricted tumor-associated antigen presentation to tumor-specific TH cells for their optimal triggering.

Indeed, beside controlling MHC-II gene expression, CIITA acts on other crucial steps of the antigen processing and presentation mechanism, via upregulation of Invariant chain [26] and DM [27] expression. Moreover, crucial to this approach were the assumptions, verified by previous elegant studies, that endogenous proteins (as most tumor antigens are) could access the MHC class II pathway of antigen presentation [28,29] and that peptides of these proteins could be recognized and serve as immunogen for TH cell triggering [30,31].

Our studies have demonstrated that efficient rejection of CIITA-transfected tumor cells of distinct histological origin can be achieved in high percentage of injected immunocompetent syngeneic mice [26,32,33]. Importantly, CIITA-tumor vaccinated mice develop an anamnestic response allowing them to reject parental tumors very efficiently [26,32,33]. That CIITA-dependent MHC class II expression in tumor cells was instrumental to trigger a protective immune response against the parental tumor was also demonstrated by vaccination experiments with non replicating CIITA-transfected tumor cells [34]. The immunological basis of tumor rejection upon vaccination with CIITA-transfected tumor cells was extensively investigated. Rejection and/or reduced tumor growth were mediated by tumor specific CD4+ TH and CD8+ CTL [26,32-34]. Importantly, tumor-specific primed TH cells were long-living memory cells and they could adoptively transfer resistance to tumor growth even after many months from original stimulation [33], implying the stabilization of a protective phenotype of effector cells over time.

The comparative study of the tumor microenvironment and of tumor draining lymph nodes in mice injected with parental or with CIITA-transfected tumor cells provided critical insight on the mechanisms triggered by CIITA-transfected tumor cells and their possible role as surrogate APCs [32]. Tumors derived from parental cells presented scarce infiltrate, represented mainly by macrophages and neutrophils, very few CD4^+^ T cells, absence of DC and CD8^+^ T cells. In contrast the site of CIITA-transfected tumor cells injection was rapidly infiltrated by CD4^+^ T cells. This was followed, days later, by the appearance of DC and CD8+ T cells and, immediately after, by the generation of extensive areas of tumor cell necrosis. The fact that CD4^+^ T cells colonized CIITA-tumor tissue before CD8^+^ T cells and DC, along with the capacity of CIITA-expressing tumor cells to process and present antigenic peptides to CD4^+^ T cells in vitro [32,35], supports the hypothesis that much of the tumor-specific TH cell triggering and/or restimulation takes place in the tumor tissue itself and is directly mediated by tumor cell-derived MHC class II molecules, as previously suggested [36-38].

Interestingly, mice rejecting CIITA-tumors and mice vaccinated with CIITA-tumor cells rejecting a challenge with parental tumors displayed a polarized CD4+ TH1 cell phenotype in their tumor-draining lymph nodes, as compared to TH2-like cells found in parental tumor-bearing mice. Moreover, the fact that CIITA-transfected tumor cells could trigger a potent anamnestic and persistent anti-tumor T cell response without an apparent sequel of autoimmunity, suggests that most of the anti-tumor response was directed against tumor- and not self-derived antigens.

The success of this approach underscores the importance of the optimal MHC-II-restricted antigen presentation, suggests that most tumor cells produce sufficient amounts of tumor antigens and renders unnecessary to know *a priori* the nature, the identity and the immunogenic hierarchy of the tumor-associated antigen(s). It is of relevance that the idea of increasing the density of tumor antigen displayed by MHC-II molecules, as it results from our approach of rendering tumor cells MHC-II positive by stable expression of CIITA, has been pursued also by other approaches based, for example, on providing sustained antigenic epitope in endosomal compartments by constructing Invariant chain-antigenic epitope chimeras [39-41] or by treating tumor cells with epigenetic modifiers that increase the expression of tumor-specific shared antigens [42,43].

## Optimal MHC-II-restricted antigen presentation to TH cells in anti-tumor therapy: the example of L19-TNFα conjugate in therapy-induced anti-tumor vaccination

In collaboration with Luciano Zardi, Laura Borsi and their team we have conducted experiments of tumor therapy in animal models by using mouse TNFα (mTNFα) covalently bound to a Fv antibody (L19) specific for the beta form of Fibronectin, selectively expressed in tumor neovasculature [44]. Injection of L19mTNFα conjugate induces a dramatic necrosis of established tumors as it allows concentrating therapeutically active doses of TNFα at the tumor level. When combined with the cytostatic drug melphalan, this treatment dramatically potentiated the effect of melphalan at the tumor site.

Two crucial observations, related to the importance of the adaptive immune response against the tumor in this approach, were made. First, the treatment resulted in a high rate of complete, long-lasting tumor eradication in distinct tumor models without any apparent adverse side effects and with no recurrence. Second, tumor-bearing immunodeficient SCID mice did not respond well to the L19mTNFα treatment even when combined with melphalan [45]. This prompted us to analyze whether the cured mice developed a tumor-specific immunity. Indeed, all cured mice were resistant to tumor challenge and the tumor rejection was mediated by CTL and, particularly, by long-living, tumor specific CD4+ TH cells [45]. TH cells were crucial for the establishment of what we defined as “therapy induced anti-tumor vaccination” because naive mice depleted of CD4+ TH cells were unable to reject primary tumors after L19mTNFα/melphalan treatment [46]. Moreover, CD4+ TH cells derived from cured animals treated with L19mTNFα/melphalan, while fully competent in generating tumor rejection when adoptively transferred together with tumor cells in naive mice, were incapable of inducing tumor rejection in CD8-depleted naïve animals, strongly suggesting that a major protective role of primed anti-tumor CD4+ T cells lies in triggering CD8+ naive T cells to become functionally mature antitumor CTL effectors [46]. Taken together, these findings strongly indicate that the L19mTNFα/melphalan treatment was instrumental in generating optimal MHC-II-restricted tumor antigen presentation to efficiently trigger tumor specific TH cells which, in turn, triggered naïve CTL precursors and sustained their cytolytic anti-tumor effector function.

Histological characterization of the tumor tissue evidenced, early after treatment, distinctive areas of necrosis with infiltration, mainly, of granulocytes and macrophages. One week after L19mTNFα/melphalan treatment a remarkable increase in the number of CD4+ and CD8+ T cells infiltrating the tumor was observed as compared to tumors from untreated animals. This was accompanied by a dramatic increase in granulocyte infiltration, to a lesser extent of macrophages and by the extension of areas of tumor necrosis [46]. Thus, the tumor necrosis induced by L19mTNFα/melphalan and the earlier infiltration of granulocytes contributing to tumor cell killing could be the key element to provide sufficient amount of tumor antigens for fuelling professional APC, which could then stimulate specific anti-tumor CD4+ TH cells and CD8+ effector CTL, leading to the complete rejection of the tumor and to the establishment of a critical reservoir of memory effector cells responsible for the accelerated rejection of the tumor upon challenge.

Further phenotypic and functional characterization of the CD4+ T cells involved in the priming of the anti-tumor immune response following therapeutic treatment revealed that while untreated tumor-bearing mice had in their spleen and tumor-draining lymph nodes IL-4-secreting TH2-type cells, treated mice displayed a mixed TH1- and TH2-type of response with a great percentage of cells secreting IFNγ [46,47]. Thus, as it was shown for the preventive vaccination approach with CIITA-expressing tumor cells, a rapid appearance and conversion, although not exclusive, toward a TH1 immune phenotype was associated with the protective adaptive anti-tumor response generated by the treatment with L19mTNFα and melphalan.

**Figure 1:**
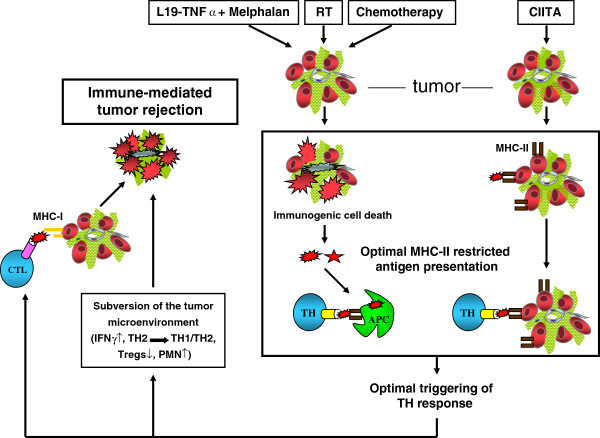
**Optimal MHC-II-restricted tumor antigen presentation as a key parameter for an effective anti-tumor adaptive immune response.** Optimal MHC-II-restricted tumor antigen presentation may result from either surrogate antigen presenting activity by tumor cells expressing CIITA-dependent MHC class II (MHC-II) molecules or by increasing the amount of tumor antigen availability for classical antigen-presenting cells (APC) via therapeutical approaches resulting in immunogenic cell death, such as radiotherapy (RT), chemotherapy, and biotherapy with, for example, L19-TNFα and melphalan (L19-TNFα + Melphalan). Optimal MHC-II-restricted tumor antigen presentation generated by the two approaches is instrumental for the optimal triggering of anti-tumor CD4+ T helper cells (TH). These MHC-II-restricted anti-tumor TH cells have a dual action in the process of immune-mediated tumor rejection: a) they are required for the activation, proliferation and cytolytic activity of CD8+ anti-tumor cytotoxic T lymphocytes (CTL); b) they are major players in the subversion of the tumor microenvironment toward an anti-tumor milieu by polarizing, for example, the lymphocyte infiltrate toward a mixed TH1/TH2 or exclusive TH1 population with an increased frequency of IFNγ secreting cells, by favouring the recruitment of inflammatory cells such as polymorphonuclear cells, by inhibiting the function and/or the recruitment of leukocytes with suppressive action on TH cells and on tumor-specific CTL.

All together the experimental evidences gathered from our approaches of either preventive or therapy–induced anti-tumor vaccination clearly demonstrate that a subversion toward an anti-tumor microenvironment can be generated by promoting optimal MHC-II-restricted antigen presentation to TH cells not only before the tumor onset but also after cancer has developed (Figure 1).

## Radiotherapy and chemotherapy as procedures to potentially induce optimal MHC-II-restricted antigen presentation to TH cells

To the light of these findings and in consideration that an optimal MHC-II-restricted tumor antigen presentation to TH cells can be obtained in different ways, a large series of previously published data on the appearance of an anti-tumor immune response following conventional anti-tumor therapy, may be re-interpreted also as a way to offer sufficient amounts of MHC-bound tumor antigens to the adaptive immune system.

For example, radiation therapy (RT) particularly when localized to the tumor mass, given alone [48] or in combination with immunostimulatory cytokines (IL-2 and TNFα) [49,50] enhances its efficacy by generating anti-tumor specific CTL responses. RT can also increase CTL stimulation by up-regulating MHC class I expression in tumor cells [51,52] and, more interestingly, by positively influencing antigen processing and presentation by dendritic cells [53].

Chemotherapy can also increase the immunogenicity of tumor cells which are recognized by immune effectors [54]. Central to this role of RT and Chemotherapy is the concept of immunogenic cell death that, provoked by the therapeutical agent, finally ends up in the triggering of an adaptive immune response [55], a concept that can be easily accommodated within the frame of the optimal MHC-II-restricted antigen presentation to TH cells described here (Figure 1).

We firmly believe that re-interpretation of the available data on the several ways through which preventive and therapeutic approaches to fight cancer may result in the generation of a protective adaptive immune response against the tumor will lead to the careful consideration that a crucial parameter underlying this effect is indeed the better availability of tumor antigens offered for recognition by tumor-specific TH via an optimal MHC-II restricted antigen presentation. This will possibly help to better understand the initial phases of the immune response against tumors and offer potential new strategies to prevent and fight cancer.

## Competing interests

The authors declare no financial/commercial conflicts of interest.

## Authors’ contribution

RSA and GT equally contributed to the manuscript by conceiving, writing, reading and approving it in its final form.
